# Another look at silent circulation of poliovirus in small populations

**DOI:** 10.1016/j.idm.2018.06.001

**Published:** 2018-06-09

**Authors:** Dominika A. Kalkowska, Radboud J. Duintjer Tebbens, Kimberly M. Thompson

**Affiliations:** Kid Risk, Inc., 605 N. High St. #253, Columbus, OH 43215, USA

**Keywords:** Polio, Small populations, Silent circulation, Stochastic modeling, AFP, acute flaccid paralysis, CFP, case-free period, CNC, confidence about no circulation, CNCx%, time when the confidence about no circulation exceeds x%, DEFP, detected-event-free period, OPV, oral poliovirus vaccine, POE, Probability of eradication, TBC, time between detected cases, TUC, time of undetected circulation after the last detected-event, TUCx%, xth percentile of the TUC, WPV, wild poliovirus

## Abstract

**Background:**

Silent circulation of polioviruses complicates the polio endgame and motivates analyses that explore the probability of undetected circulation for different scenarios. A recent analysis suggested a relatively high probability of unusually long silent circulation of polioviruses in small populations (defined as 10,000 people or smaller).

**Methods:**

We independently replicated the simple, hypothetical model by Vallejo et al. (2017) and repeated their analyses to explore the model behavior, interpretation of the results, and implications of simplifying assumptions.

**Results:**

We found a similar trend of increasing times between detected cases with increasing basic reproduction number (R_0_) and population size. However, we found substantially lower estimates of the probability of at least 3 years between successive polio cases than they reported, which appear more consistent with the prior literature. While small and isolated populations may sustain prolonged silent circulation, our reanalysis suggests that the existing rule of thumb of less than a 5% chance of 3 or more years of undetected circulation with perfect surveillance holds for most conditions of the model used by Vallejo et al. and most realistic conditions.

**Conclusions:**

Avoiding gaps in surveillance remains critical to declaring wild poliovirus elimination with high confidence as soon as possible after the last detected poliovirus, but concern about transmission in small populations with adequate surveillance should not significantly change the criteria for the certification of wild polioviruses.

## 1 Introduction

The detection of a wild poliovirus (WPV) in Borno state in Northeast Nigeria in 2016 (Nnadi et al., 2017), at a time when many hoped WPV circulation had stopped in Africa, raised questions about the possibility of prolonged undetected poliovirus circulation in small populations. The serotype 1 WPV detected in 2016 in Borno occurred almost two years after the prior most recent case in Nigeria in 2014, although it likely originated from a distinct lineage last detected in Borno in 2013 (Nnadi et al., 2017). Due to civil unrest, polio eradication efforts could not reach parts of Borno with vaccination or surveillance for several years, and some areas remain inaccessible (Nnadi et al., 2017). Similar resurfacing of a WPV after multiple years without detection previously occurred in the Sudan and Chad (World Health Organization, 2005a, 2005b), and surveillance continues to occasionally detect WPVs with no closely linked ancestors in Pakistan and Afghanistan (i.e., orphan viruses) (Cowger et al., 2017). Poliovirus surveillance primarily relies on the detection of symptomatic cases of acute flaccid paralysis (AFP) and tests of their stool for the presence of poliovirus. However, only less than 1% of WPV infections in susceptible individuals result in paralytic symptoms (i.e., approximately 1/200, 1/2,000, and 1/1,000 for WPV serotypes 1, 2, and 3, respectively) (Nathanson & Kew, 2010). This implies that hundreds to thousands of infections can occur between successive cases, leading to a situation of silent circulation. While all documented instances of prolonged undetected circulation occurred in the context of likely gaps in surveillance, the possibility of silent poliovirus circulation contributes to the ability of transmission to go undetected.

Motivated by the re-emergence in Borno, a recent study used a stochastic model to examine whether simple, small, hypothetical populations not reached at all by vaccination can perpetuate WPV transmission while experiencing very long intervals between polio cases (Vallejo, Keesling, Koopman, & Singer, 2017). The potential for silent circulation influences the confidence about the interruption of poliovirus transmission as a function of time after the last detected poliovirus, which informs the decision to certify wild poliovirus eradication in a country, region, and globally. Global certification of a wild poliovirus serotype affects when the world can coordinate the cessation of the use of homotypic oral poliovirus vaccine (OPV), which represents a necessary step to end all paralytic poliomyelitis disease (polio) caused by polioviruses, because OPV itself can in rare instances cause polio (Duintjer Tebbens et al., 2006; World Health Organization, 2005a, 2005b, 2013). Delaying OPV cessation leads to substantial additional costs (Duintjer Tebbens et al., 2011), but premature OPV cessation implies a risk of a WPV reemergence that could become very difficult to control. As of early 2018, of the six World Health Organizations (WHO) regions, only the African and the Eastern Mediterranean regions remain not certified as polio-free due to the possibility of continued WPV transmission in Borno (African region) and continuing polio cases in Pakistan and Afghanistan (Eastern Mediterranean region) (World Health Organization, 2018). Moreover, of the three WPV serotypes, the world certified serotype 2 WPV eradication in 2015 (Global Polio Eradication Initiative, 2015) and did not report any polio cases due to serotype 3 WPV since 2012 (Kew et al., 2014), with only serotype 1 WPV continuing to cause reported polio cases.

Based on epidemiological experience and modeling, certifying a region or the world as polio-free requires no observed polio cases for at least three years (Debanne & Rowland, 1998; Eichner & Dietz, 1996; Kalkowska, Duintjer Tebbens, Pallansch, et al., 2015; World Health Organization, 2017). Prior to Vallejo et al. (2017), numerous modeling studies addressed the possibility of prolonged poliovirus circulation without any detected polio cases in different populations using different metrics, and spanning different model structures and assumptions about surveillance, vaccination, transmissibility, and demographics (Berchenko et al., 2017; Eichner & Dietz, 1996; Famulare, 2016; Houy, 2015; Kalkowska, Duintjer Tebbens, Pallansch, et al., 2015; Kalkowska, Duintjer Tebbens, & Thompson, 2012). These models generally agree that in most realistic situations, 3 years without any detected cases implies at least 95% confidence about no circulation, although our studies show that results depend on surveillance quality, serotype, seasonality, and vaccination strategies (Kalkowska, Duintjer Tebbens, Pallansch, et al., 2015; Kalkowska et al., 2012). In contrast to the prior literature, Vallejo et al. (2017) prominently reported (in their abstract) a 22% chance of at least 3 years between successive polio cases in a population of 10,000 people despite a 100% case detection rate. In a correction, Vallejo, Keesling, Koopman, and Singer (2018) report an error in the calculation of the average age of infection that resulted in the use of unrealistically high values for the basic reproduction number (R_0_), which led them to note the limited applicability of their findings to real-world situations. Nonetheless, the potential use of their findings in deliberations about polio certification motivated us to replicate the Vallejo et al. (2017) analyses to take a closer look at the model behavior, the interpretation of the results (particularly in the context of the relevant prior literature that they did not consider), and the implications of some of their simplifying assumptions. Although they issued a correction that highlighted one unrealistic model input assumption (Vallejo et al., 2018), we highlight multiple other attributes that they did not consider that further limit the utility of their approach and model for supporting policy, including the unrealistic assumptions about the existence of completely isolated small populations that remain at the endemic equilibrium with no vaccination and yet benefit from perfect surveillance.

## 2 Material and methods

We used the model structure, notation, input values, and microsimulation algorithm descriptions provided by Vallejo et al. (2017) to reconstruct their model in Java using the Eclipse open development platform (Eclipse Foundation, Inc., Ottawa, Ontario, Canada). The first two authors independently reconstructed the model. The model assumes a constant population (i.e., birth rate = death rate) distributed over five compartments: susceptible (i.e., never infected), first infection (susceptible to paralysis), temporary full immunity to infection after recovery from infection, partial susceptibility to infection but not paralysis after waning, and reinfection of partially susceptible individuals. The paralysis-to-infection ratio determines the probability of a first infection leading to a polio case, and the detection rate determines the probability that surveillance would detect a polio case. The model ignores the 1–3-day latent period between virus exposure and becoming infectious to others (Duintjer Tebbens, Pallansch, Kalkowska, et al., 2013), and characterizes the infectious period using a single stage, which implies an exponential recovery process with an unrealistically long tail (Duintjer Tebbens, Pallansch, Kalkowska, et al., 2013; Lloyd, 2001). The model uses the Gillespie algorithm (Gillespie, 1976), which randomly generates when transition events between the compartments occur. Consequently, each stochastic iteration of the model results in different event times. This leads to difficulties in interpretation of the results in Figures 5 and 6 from Vallejo et al. (2017) that they presented as a function of “event steps,” (Vallejo et al., 2017) and therefore we provided the corresponding results also as a function of time, which provides a more natural interpretation.

Briefly, Vallejo et al. (2017) explored the model behavior in terms of time between detected cases (TBC), which represents a different metric than those developed in the prior literature (Berchenko et al., 2017; Eichner & Dietz, 1996; Famulare, 2016; Houy, 2015; Kalkowska, Duintjer Tebbens, Pallansch, et al., 2015; Kalkowska et al., 2012). Vallejo et al. (2017) focused on the proportion of stochastic iterations of their model in which any TBC exceeded 3 years. They varied the detection rate, the population size, the basic reproduction number (R_0_), and the pattern of waning immunity (i.e., “fast-shallow,” “intermediate,” or “slow-deep” (Koopman et al., 2017), which considered the same R_0_ values, and which Vallejo et al. (2018) identified as unrealistic). Vallejo et al. (2017) also kept the paralysis-to-infection ratio constant at 0.005, which corresponds to the consensus estimate for serotype 1 WPV (Duintjer Tebbens, Pallansch, Kalkowska, et al., 2013; Nathanson & Kew, 2010). They initialized their simulations at the endemic equilibrium of the related differential equation based (DEB) model and reported results based on a single simulation run of 1,000 stochastic iterations. We emphasize that for polio, the assumption of a small, isolated population with no immunization that starts at the endemic equilibrium with perfect surveillance represents an unrealistic situation (i.e., in reality, such a population cannot indigenously sustain persistent transmission), but for purposes of this analysis we replicated the Vallejo et al. (2017) model as published.

For each scenario (i.e., defined as a set of assumed values for population size, R_0_, waning immunity pattern (Koopman et al., 2017), and detection rate), we ran the corresponding DEB model for a period of 100 years to reach its endemic equilibrium. Using this equilibrium, rounded to natural numbers, as the initial condition for the microsimulation model, we ran each stochastic iteration for 15 years or until infection died out, whichever came first. Instead of just running a single simulation and assuming that it represented the truth, we recognized the stochastic nature of the model and the potential for variability in the results as a function of the number of iterations. To characterize the stochastic variability as a function of number of iterations in the simulation, we explored the stochastic variability in the results by performing multiple simulation runs of different numbers of iterations (n_i_). We recorded the variation around the mean results of n_i_ = 1,000, 10,000, or 100,000 iterations by running n_s_ = 10 simulations for each n_i_ to demonstrate the range of results possible with a single simulation run of n_i_ iterations. We considered the scenarios of a population (N) of 3,500 or 10,000, R_0_ equals 10, a 100% detection rate, and all three patterns of waning immunity. Due to the relative instability we found in the results with relatively small numbers of iterations (i.e., n_i_ = 1,000), we reproduced the Vallejo et al. (2017) analyses with 100,000 iterations.

We also explored different ways to interpret the results. Notably, the TBC metric informs the probability that consecutive detected cases occur at least 3 years apart in a stochastic iteration, given the detection of cases. Put differently, it answers the question: “given that we know some transmission is occurring in a small population, what is the probability of observing at least 3 years between successive (detected) cases?” However, a more relevant policy question is: “for a small population that we know supported transmission initially, what is the probability that we would observe a gap of at least 3 years between cases?” Answering the latter question requires dividing the number of iterations with an occurrence of a TBC of at least 3 years by the total number of stochastic iterations performed (instead of only those with at least two detected cases). Neither metric directly informs the question of interest for decision makers: “given that we haven't observed any cases for X amount of time, what is the probability that circulation continues?” The prior literature focused on answering this question. Characterizing the probability of circulation still occurring given 3 years without a detected case requires direct comparison of the frequency of 3 years without a detected case occurring despite continued transmission with the frequency of 3 years without a detected case when transmission truly stopped (as recognized by the seminal work by Eichner & Dietz, 1996). Prior studies used the following more appropriate metrics for measuring the confidence about no circulation of poliovirus transmission (Eichner & Dietz, 1996; Kalkowska, Duintjer Tebbens, Pallansch, et al., 2015; Kalkowska et al., 2012):

• POE – “the probability of eradication defined as the fraction of stochastic iterations in which die-out occurs (i.e., prevalence below the transmission threshold)”
• DEFP – “the detected-event-free period defined as the time in months since the last detected case”
• CNC – “confidence about no circulation given the DEFP approximated as (1 - the number of DEFPs equal to t months with ongoing WPV circulation, divided by all DEFPs of t months)”
• CNCx% – “the time when the confidence about no circulation exceeds x% (i.e., CNC95%, CNC99%)”
• TUC – “the time of undetected circulation after the last detected-event (for those iterations in which extinction occurs)”
• TUCx% – “the xth percentile of the TUC (i.e., TUC95%, TUC99%)” (Kalkowska, Duintjer Tebbens, Pallansch, et al., 2015).

We note that models that only considered the occurrence of cases regardless of poliovirus detection (Eichner & Dietz, 1996; Kalkowska et al., 2012) used a case-free period (CFP) instead of the DEFP. Subsequent modeling extended the concept of the CFP to the DEFP to allow for the detection of transmission in environmental surveillance, because acute flaccid paralysis surveillance may not detect all cases and environmental surveillance can detect the presence of transmission without cases (Kalkowska, Duintjer Tebbens, Pallansch, et al., 2015; Kalkowska, Duintjer Tebbens, Pallansch, & Thompson, 2018). The estimation process evaluates the DEFP and CNC discretely using a time step of months.

Finally, while the assumed mean infectious period of 28 days appears realistic based on the evidence and expert opinion (Duintjer Tebbens et al., 2013; Duintjer Tebbens et al., 2013), the single-stage process implies unrealistic proportions of infected individuals of 34%, 12%, and 4% remaining infectious to others at 1, 2, or 3 months, respectively. Recognizing the issues associated with the use of a single stage infection process (Duintjer Tebbens, Pallansch, Kalkowska, et al., 2013; Lloyd, 2001), we explored the impact of breaking the infection process into either 2 or 4 even stages, which effectively narrows the distribution of the infectious period while maintaining the same mean infectious period.

## 3 Results

**Fig. 1:**
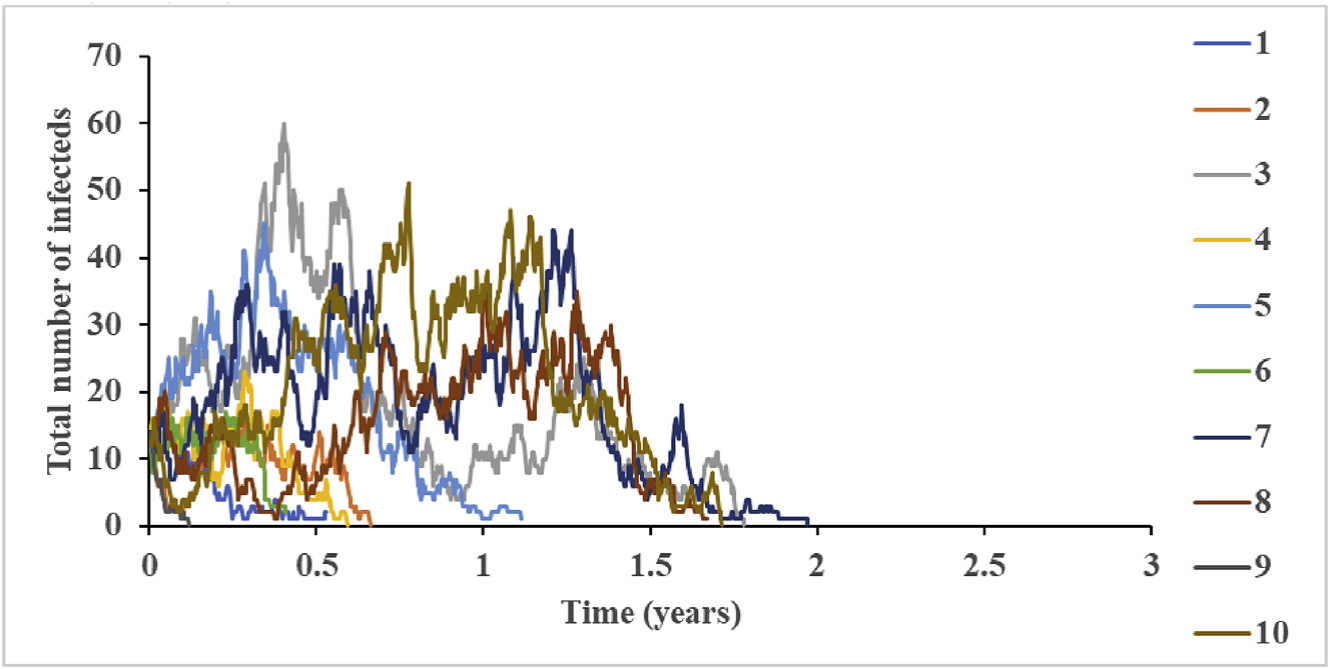
The variation in the total infected population over time based on 10 iterations for R_0_ = 10, N = 3,500, and fast-shallow waning.

Fig. 1 shows the total number of infected individuals as a function of time for 10 different stochastic iterations of one model scenario (i.e., R_0_ = 10, N = 3,500, and fast-shallow waning). We show 10 iterations to demonstrate the substantial variation between iterations in both the intensity of transmission and the time until elimination of the virus. With all stochastic models, the number of iterations performed will determine the stability of the results, with larger numbers of iterations required to capture the model behavior by averaging over all iterations.

**Fig. 2:**
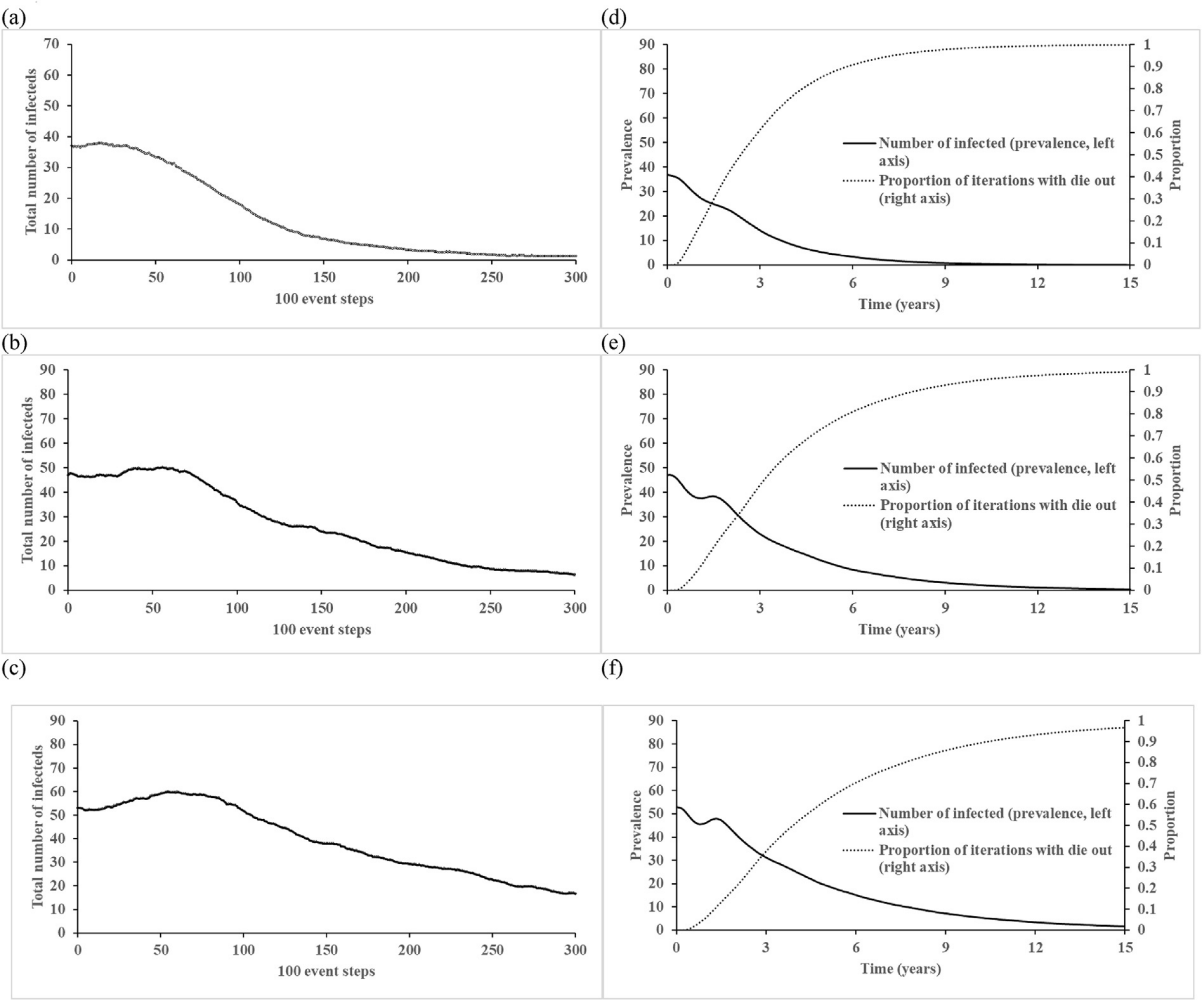
The count of the total infected population for N = 10,000 with fast-shallow waning immunity dynamics, and: (a) R_0_ = 10, (b) R_0_ = 15, (c) R_0_ = 20 averaged over n_i_ = 1,000 at every 100 event steps and (d) R_0_ = 10, (e) R_0_ = 15, (f) R_0_ = 20 averaged over n_i_ = 100,000 over time.

**Fig. 3:**
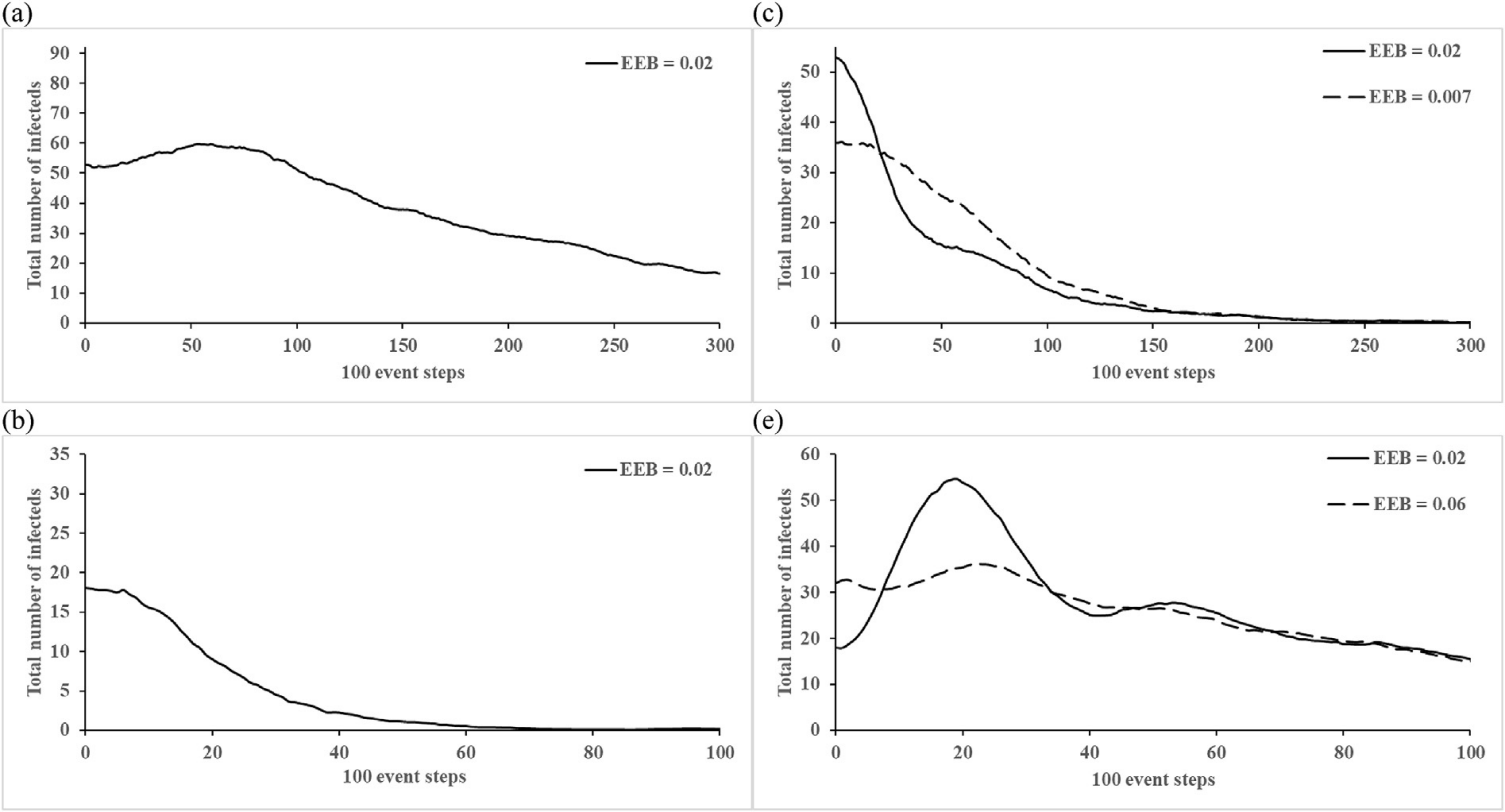
The effect of modifying the birth rate on the count of the total number of infected individuals in the populations with R_0_ = 20 and fast-shallow waning immunity dynamics: (a) N = 10,000, birth rate = 0.02; (b) N = 3,500, birth rate = 0.02; (c) N = 10,000, birth rates = 0.007 and 0.02 (used by Vallejo et al.); (d) N = 3,500, birth rates = 0.06 and 0.02 (used by Vallejo et al.) averaged over n_i_ = 1,000 at every 100 event steps. Abbreviations: EEB, endemic equilibrium birth rate.

Table 1 reports our attempt to replicate the summary statistics for TBC distributions for different scenarios presented in Figs. 2 and 3 of the Vallejo et al. (2017) paper using a single simulation of 100,000 iterations. Our results show systematically lower results (shorter TBCs) for the interquartile ranges.

**Table 1:** The distribution of time (years) between detected polio cases for different population sizes, R_0_ values, and waning immunity patterns (Koopman et al., 2017) (based on n_i_ = 100,000). Compare to Figures 1 and 2 in Vallejo et al. (2017).

Population Size		3,500					10,000				
R_0_	Waning Pattern	min	Q1	median	Q3	max	min	Q1	median	Q3	max
*Detection Rate*		*100%*									
10	fast-shallow	0.00	0.13	0.31	0.63	4.50	0.00	0.15	0.39	0.90	7.51
	intermediate	0.00	0.14	0.32	0.67	5.22	0.00	0.15	0.40	0.91	8.64
	slow-deep	0.00	0.13	0.33	0.68	4.91	0.00	0.16	0.40	0.93	9.31
15	fast-shallow	0.00	0.14	0.35	0.75	4.91	0.00	0.16	0.44	1.06	9.84
	intermediate	0.00	0.14	0.34	0.73	5.41	0.00	0.16	0.43	1.02	10.60
	slow-deep	0.00	0.14	0.35	0.74	5.05	0.00	0.16	0.43	1.03	9.45
20	fast-shallow	0.00	0.16	0.40	0.88	6.69	0.00	0.17	0.49	1.18	11.92
	intermediate	0.00	0.14	0.37	0.82	5.94	0.00	0.17	0.46	1.10	10.74
	slow-deep	0.00	0.14	0.37	0.80	6.66	0.00	0.17	0.46	1.10	10.52
*Detection Rate*		*75%*									
10	fast-shallow	0.00	0.13	0.32	0.65	5.86	0.00	0.18	0.47	1.10	10.70
	intermediate	0.00	0.14	0.34	0.69	4.28	0.00	0.19	0.49	1.13	10.70
	slow-deep	0.00	0.14	0.35	0.74	4.71	0.00	0.19	0.50	1.15	11.92
15	fast-shallow	0.00	0.15	0.37	0.81	6.15	0.00	0.20	0.56	1.34	11.72
	intermediate	0.00	0.15	0.37	0.80	5.68	0.00	0.20	0.54	1.28	12.71
	slow-deep	0.00	0.15	0.38	0.82	6.17	0.00	0.20	0.54	1.29	11.25
20	fast-shallow	0.00	0.17	0.44	0.96	6.83	0.00	0.22	0.64	1.47	10.78
	intermediate	0.00	0.16	0.39	0.86	5.98	0.00	0.21	0.58	1.37	12.51
	slow-deep	0.00	0.15	0.39	0.84	6.03	0.00	0.21	0.58	1.38	13.84
*Detection Rate*		*50%*									
10	fast-shallow	0.00	0.15	0.36	0.73	3.73	0.00	0.23	0.61	1.42	11.47
	intermediate	0.00	0.14	0.37	0.75	4.72	0.00	0.24	0.64	1.48	11.81
	slow-deep	0.00	0.15	0.38	0.79	4.98	0.00	0.24	0.64	1.51	13.70
15	fast-shallow	0.00	0.17	0.41	0.87	5.17	0.00	0.27	0.76	1.73	14.31
	intermediate	0.00	0.15	0.37	0.76	5.12	0.00	0.26	0.71	1.67	13.22
	slow-deep	0.00	0.16	0.38	0.80	5.17	0.00	0.26	0.73	1.70	12.24
20	fast-shallow	0.00	0.17	0.44	0.98	5.86	0.00	0.30	0.91	1.90	13.80
	intermediate	0.00	0.17	0.41	0.90	5.92	0.00	0.27	0.80	1.78	14.56
	slow-deep	0.00	0.17	0.43	0.90	5.20	0.00	0.28	0.81	1.79	14.41
*Detection Rate*		*25%*									
10	fast-shallow	0.00	0.16	0.37	0.80	3.30	0.00	0.31	0.83	1.93	13.67
	intermediate	0.00	0.16	0.38	0.84	3.96	0.00	0.33	0.89	2.03	14.10
	slow-deep	0.00	0.17	0.40	0.83	6.68	0.00	0.33	0.90	2.08	13.95
15	fast-shallow	0.00	0.15	0.39	0.91	3.87	0.00	0.39	1.18	2.41	14.00
	intermediate	0.00	0.18	0.38	0.88	4.08	0.00	0.37	1.09	2.32	14.24
	slow-deep	0.00	0.16	0.43	0.90	4.08	0.00	0.36	1.07	2.30	13.31
20	fast-shallow	0.00	0.19	0.46	0.99	4.79	0.00	0.48	1.43	2.72	14.77
	intermediate	0.00	0.17	0.41	0.98	7.37	0.00	0.41	1.25	2.48	13.33
	slow-deep	0.00	0.19	0.49	0.97	5.39	0.00	0.41	1.23	2.47	14.64

Abbreviations: max, maximum; min, minimum; Q1, 25th percentile; Q3, 75th percentile; R_0_, basic reproduction number.

Table 2 summarizes the results of our analysis of the main results highlighted by Vallejo et al. (2017). We note that they report (in their Tables 3 and 4) one metric as a proportion and one metric as a percentage, which we replicate while noting that this requires the reader to multiply or divide by 100 to get the metrics on the same scale. To facilitate direct comparison, the first two columns of our Table 2 show the results as reported in Table 3 of Vallejo et al. (2017) of (i) the proportions of all iterations with more than 1 detected polio case (“>1 case/n_i_”) and (ii) the percent of iterations with more than 1 detected polio case for which any TBC exceeded 3 years (“>3 years/>1 case”). As mentioned in the methods section we recognized that the second of these metrics provided inflated estimates of the total probability of observing TBCs of greater than 3 years in the entire simulation given the conditioning on observing more than one case. Specifically, the “>3 years/>1 case” metric alone does not account for the possibility that polio cases may not occur in the first place, and it only provides information about the probability that we may detect silent circulation after more than 3 years in the case that we know that silent circulation is ongoing, which we would not know (and which makes this not a metric of interest to policy makers). Consequently, we added a more informative metric (shown in bold in Table 2, Table 3, Table 4) that reports the percent of all iterations for which any TBC exceeded 3 years (“>3 years/n_i_”). We inferred the value of this metric for the Vallejo et al. (2017) results from the reported results.

**Table 2:** The influence of the sample size on of the proportions of all iterations with more than 1 detected case (“>1 case/n_i_”), the percent of iterations with more than 1 detected case and time between detected cases greater than 3 years (“>3 years/>1 case”), and the percent of all iterations with time between detected cases greater than 3 years (“>3 years/n_i_”) for different population sizes (i.e., N = 3,500 and 10,000), R_0_ = 10, all three waning immunity patterns (Koopman et al., 2017), and 100% detection rate.

n_s_, n_i_	n_s_ = 1, n_i_ = 1,000			n_s_ = 10, n_i_ = 1,000			n_s_ = 10, n_i_ = 10,000			n_s_ = 10, n_i_ = 100,000		
	>1 case/n_i_	>3 yrs/>1 case	**>3 yrs/n**_i_	>1 case/n_i_	>3 yrs/>1 case	**>3 yrs/n**_i_	>1 case/n_i_	>3 yrs/>1 case	**>3 yrs/n**_i_	>1 case/n_i_	>3 yrs/>1 case	**>3 yrs/n**_i_
	Vallejo et al. (2017)			mean (SD)	mean (SD)	**mean (SD)**	mean (SD)	mean (SD)	**mean (SD)**	mean (SD)	mean (SD)	**mean (SD)**
*Population Size*	*N* = *3500*											
fast-shallow	0.064	6.25%	**0.40%**	0.058 (0.005)	0.73% (0.89%)	**0.04% (0.05%)**	0.061 (0.002)	0.41% (0.20%)	**0.03% (0.01%)**	0.061 (0.001)	0.38% (0.05%)	**0.02% (0.00%)**
intermediate	0.062	8.06%	**0.50%**	0.064 (0.009)	0.49% (0.75%)	**0.03% (0.05%)**	0.062 (0.003)	0.47% (0.30%)	**0.03% (0.02%)**	0.064 (0.001)	0.52% (0.07%)	**0.03% (0.00%)**
slow-deep	0.076	2.62%	**0.20%**	0.068 (0.008)	0.31% (0.62%)	**0.02% (0.04%)**	0.067 (0.003)	0.59% (0.28%)	**0.04% (0.02%)**	0.067 (0.001)	0.49% (0.08%)	**0.04% (0.01%)**
*Population Size*	*N* = *10,000*											
fast-shallow	0.578	12.98%	**7.50%**	0.560 (0.014)	4.74% (0.74%)	**2.65% (0.41%)**	0.556 (0.007)	5.11% (0.27%)	**2.84% (0.12%)**	0.558 (0.001)	5.09% (0.08%)	**2.84% (0.05%)**
intermediate	0.603	13.76%	**8.30%**	0.575 (0.014)	5.76% (0.57%)	**3.31% (0.36%)**	0.584 (0.004)	5.88% (0.28%)	**3.44% (0.16%)**	0.585 (0.002)	5.85% (0.07%)	**3.42% (0.05%)**
slow-deep	0.597	13.40%	**8.00%**	0.607 (0.019)	6.13% (1.02%)	**3.72% (0.61%)**	0.597 (0.005)	6.02% (0.25%)	**3.60% (0.15%)**	0.596 (0.002)	6.30% (0.10%)	**3.75% (0.06%)**

Abbreviations: n_s_, number of simulations; n_i_, number of iterations; SD, standard deviation; yrs, years.

Our results shown in Table 2 report the mean values and standard deviations (SDs) that we obtained by running 10 simulations of 1,000, 10,000, or 100,000 iterations. We find similar estimates for the “>1 case/n_i_” results reported by Vallejo et al. (2017), but significantly lower values for the “>3 years/1 case” and “>3 years/n_i_” results. As noted in the methods, substantial variability can arise from stochastic iterations (e.g., Fig. 1), and this can lead to unstable estimates of relatively rare outcomes from the simulations (e.g., a TBC>3 years). Vallejo et al. (2017) did not consider stochastic variability or explore the importance of running a sufficient number of iterations to obtain stable results (i.e., they used n_s_ = 1, n_i_ = 1,000). Comparison of our results in the right columns of Table 2 to the Vallejo et al. (2017) results in the left columns show substantial stochastic variation around the means based on 1,000 stochastic iterations, especially for the “>3 years/>1 case” and the “>3 years/n_i_” metrics, which often involve very small numbers of iterations with a TBC exceeding 3 years. For example, the typical variation around the mean of the “>3 years/>1 case” metric for a population size of 3,500 exceeds the value of the mean, suggesting a long right tail of the distribution of this metric due to a bound of zero at the low end. Consistent with the central limit theorem (Durrett, 2010), increasing the number of iterations by a factor of 100 to 100,000 lowers the standard deviations roughly by a factor of 10. Given the relatively rare nature of the outcomes of interest for the TBC and other metrics associated with undetected circulation, we suggest that the results from Table 2 indicate the need for more than 1,000 iterations.

Table 3 recreates the results from Tables 3 and 4 reported by Vallejo et al. (2017). We found no significant difference between the proportions of all iterations with more than 1 detected polio case, as expected based on Table 1, Table 2. However, we found between 2 and 16 times lower percentages of iterations with a TBC exceeding 3 years given two or more detected polio cases, compared to Vallejo et al. (2017), depending on the scenario. The maximum probability of 22% reported by Vallejo et al. (2017) of at least 3 years between successive polio cases given >1 case in a population of 10,000 people despite a 100% case detection rate decreased to 12% in our independent replication of the model (see Table 3). We emphasize that this change is independent of the correction made by Vallejo et al. (2018), which highlighted the unrealistic nature of an R_0_ of 20. Moreover, we emphasize that this metric only considers those iterations in which two or more polio cases occur, which represent a subset of all the possible realizations of endemic transmission that the model produces. If we instead focus on the more appropriate percentage of all iterations for which any TBC exceeds 3 years (representing all possible realizations of what could happen in the model), then our finding suggests a maximum probability of only 9% that more than 3 years go by between successive cases (Table 3, N = 10,000, R_0_ = 20, fast-shallow waning, 100% detection, last column). We also believe that Vallejo et al. (2017) omit the silent circulation from the start of simulation until the first case, which matters particularly when only 1 case occurs and is relevant to the question of silent circulation. Notably, if we count those iterations in which more than 3 years went by until the first case then this leads to a maximum probability of 11.5% (N = 10,000, R_0_ = 20, fast-shallow waning, not shown).

**Table 3:** The proportion of iterations with more than 1 detected case (“>1 case/n_i_”), the percent of iterations with more than 1 detected case and time between detected cases greater than 3 years (“>3 years/>1 case”), and the percent of all iterations with time between detected cases greater than 3 years (“>3 years/n_i_”) for different population sizes, R_0_ values, and patterns of waning immunity patterns (Koopman et al., 2017) with 100% and 50% detection rates (based on n_i_ = 100,000).

Population Size (N)		3,500			5,000			7,000			10,000		
*Detection Rate*		*100%*											
R_0_	Waning Pattern	>1 case/n_i_	>3 yrs/>1 case	>3 yrs/n_i_	>1 case/n_i_	>3 yrs/>1 case	>3 yrs/n_i_	>1 case/n_i_	>3 yrs/>1 case	>3 yrs/n_i_	>1 case/n_i_	>3 yrs/>1 case	>3 yrs/n_i_
10	fast-shallow	0.062	0.39%	**0.02%**	0.146	1.33%	**0.19%**	0.308	3.15%	**0.97%**	0.558	5.14%	**2.87%**
	Intermediate	0.064	0.65%	**0.04%**	0.158	1.89%	**0.30%**	0.329	3.49%	**1.15%**	0.585	5.81%	**3.40%**
	slow-deep	0.067	0.48%	**0.03%**	0.161	1.99%	**0.32%**	0.337	4.14%	**1.39%**	0.594	6.36%	**3.78%**
15	fast-shallow	0.066	0.82%	**0.05%**	0.178	2.69%	**0.48%**	0.389	5.67%	**2.21%**	0.673	8.06%	**5.42%**
	Intermediate	0.064	0.70%	**0.04%**	0.171	2.53%	**0.43%**	0.372	4.96%	**1.85%**	0.654	7.25%	**4.74%**
	slow-deep	0.066	0.87%	**0.06%**	0.176	2.73%	**0.48%**	0.371	5.35%	**1.99%**	0.652	7.49%	**4.88%**
20	fast-shallow	0.077	1.42%	**0.11%**	0.211	4.46%	**0.94%**	0.451	8.49%	**3.83%**	0.747	12.08%	**9.03%**
	Intermediate	0.066	1.06%	**0.07%**	0.181	2.88%	**0.52%**	0.405	6.08%	**2.46%**	0.690	8.72%	**6.02%**
	slow-deep	0.068	1.28%	**0.09%**	0.187	3.25%	**0.61%**	0.401	6.10%	**2.45%**	0.692	8.92%	**6.17%**
*Detection Rate*		*50%*											
10	fast-shallow	0.020	0.61%	**0.01%**	0.056	2.08%	**0.12%**	0.140	6.16%	**0.86%**	0.336	11.95%	**4.02%**
	Intermediate	0.020	0.75%	**0.02%**	0.059	2.72%	**0.16%**	0.155	7.17%	**1.11%**	0.358	13.68%	**4.90%**
	slow-deep	0.021	1.27%	**0.03%**	0.059	3.12%	**0.19%**	0.159	7.43%	**1.18%**	0.372	14.51%	**5.39%**
15	fast-shallow	0.021	1.13%	**0.02%**	0.068	4.65%	**0.31%**	0.188	10.74%	**2.02%**	0.441	18.96%	**8.36%**
	Intermediate	0.021	0.78%	**0.02%**	0.065	4.12%	**0.27%**	0.179	9.86%	**1.76%**	0.417	17.36%	**7.24%**
	slow-deep	0.020	0.96%	**0.02%**	0.067	4.17%	**0.28%**	0.182	9.66%	**1.76%**	0.421	17.87%	**7.53%**
20	fast-shallow	0.024	2.07%	**0.05%**	0.085	6.91%	**0.58%**	0.239	14.93%	**3.56%**	0.533	27.00%	**14.38%**
	Intermediate	0.021	1.36%	**0.03%**	0.072	5.26%	**0.38%**	0.202	11.66%	**2.35%**	0.464	20.81%	**9.65%**
	slow-deep	0.021	1.35%	**0.03%**	0.071	5.51%	**0.39%**	0.200	11.71%	**2.35%**	0.469	21.23%	**9.95%**

Abbreviations: n_i_, number of iterations; yrs, years.

Table 4 highlights the ambiguity about the appropriate probabilistic interpretation of the TBC-based metrics and the omission of information about how many iterations exhibit persistent transmission for 15 years by providing comparisons to a more comprehensive set of metrics, based on prior research (Eichner & Dietz, 1996; Kalkowska, Duintjer Tebbens, Pallansch, et al., 2015; Kalkowska et al., 2012). The metrics from the prior literature represent a better and more appropriate characterization of the outcome of interest to decision makers because they reflect the actual situation by looking at the probability of transmission continuing given the observation of no cases as a function of time. While it may seem subtle, this differs significantly from the TBC. Table 4 shows the results of the POE, CNC95%, CNC99%, TUC95%, and TUC99% for comparison to the Vallejo et al. (2017) metrics and estimates for N = 3,500 or 10,000 with realistic R_0_ = 10. For all patterns of waning immunity, the model predicts POE = 100% for N = 3,500 and suggests time periods of 2.25 (3.08) years or less without polio cases required to achieve 95% (99%) confidence in the interruption of transmission. Also, the time of undetected circulation from the last detected case until die-out does not exceed 2.07 (2.99) years with 95% (99%) certainty. Increasing the population size (N = 10,000) decreases the POE by less than 1%, while the CNCs and TUCs rise to 3.42 and 2.57 years or less at the 95% confidence level. Table 4 also shows how the results change with the use of a 2- or 4-stage infection process. Applying a more realistic distribution for the infectious period increases the POE to 100% for the larger population (i.e., N = 10,000), while reducing the time to reach 95% (99%) confidence about no circulation by up to 10 months (13 months).

**Table 4:** The proportion of iterations with more than 1 detected case (“>1 cases/n_i_”), the percent of iterations with more than 1 detected case and time between detected cases greater than 3 years (“>3 years/>1 cases”), the percent of all iterations with time between detected cases greater than 3 years (“>3 years/n_i_”), POE, CNC95%, CNC99%, TUC95%, and TUC99% for different population sizes, R_0_ = 10, varying waning immunity patterns (Koopman et al., 2017), and 100% detection rate (based on n_i_ = 100,000).

Infectious period	Waning Pattern	Vallejo et al. (2017)			>1 case/n_i_	>3 yrs/>1 case	>3 yrs/n_i_	POE	CNC95%	CNC99%	TUC95%	TUC99%
		>1 case/n_i_	>3 yrs/>1 case	>3 yrs/n_i_								
*Population Size*		*N* = *3,500*										
1-stage infectious period	fast-shallow	0.064	6.25%	**0.40%**	0.062	0.39%	**0.02%**	100.00%	2.08	2.92	1.95	2.79
	intermediate	0.062	8.06%	**0.50%**	0.064	0.65%	**0.04%**	100.00%	2.17	3.08	2.02	2.92
	slow-deep	0.076	2.62%	**0.20%**	0.067	0.48%	**0.03%**	100.00%	2.25	3.08	2.07	2.99
2-stage infectious period	fast-shallow	–	–	**-**	0.045	0.07%	**0.00%**	100.00%	1.67	2.33	1.56	2.23
	intermediate	–	–	**-**	0.046	0.13%	**0.01%**	100.00%	1.75	2.42	1.62	2.30
	slow-deep	–	–	**-**	0.051	0.16%	**0.01%**	100.00%	1.75	2.50	1.65	2.36
4-stage infectious period	fast-shallow	–	–	**-**	0.030	0.00%	**0.00%**	100.00%	1.42	1.92	1.31	1.86
	intermediate	–	–	**-**	0.031	0.03%	**0.00%**	100.00%	1.42	2.00	1.36	1.94
	slow-deep	–	–	**-**	0.035	0.06%	**0.00%**	100.00%	1.50	2.08	1.39	2.00
*Population Size*		*N* = *10,000*										
1-stage infectious period	fast-shallow	0.578	12.98%	**7.50%**	0.558	5.14%	**2.87%**	99.88%	3.25	4.33	2.47	3.63
	intermediate	0.603	13.76%	**8.30%**	0.585	5.81%	**3.40%**	99.79%	3.33	4.50	2.55	3.76
	slow-deep	0.597	13.40%	**8.00%**	0.594	6.36%	**3.78%**	99.74%	3.42	4.58	2.57	3.82
2-stage infectious period	fast-shallow	–	–	**-**	0.455	2.13%	**0.97%**	99.99%	2.75	3.75	2.19	3.25
	intermediate	–	–	**-**	0.480	2.55%	**1.22%**	99.99%	2.83	3.93	2.26	3.31
	slow-deep	–	–	**-**	0.489	2.89%	**1.41%**	99.99%	2.92	3.92	2.27	3.37
4-stage infectious period	fast-shallow	–	–	**-**	0.394	1.15%	**0.45%**	100.00%	2.42	3.33	1.96	2.90
	intermediate	–	–	**-**	0.414	1.41%	**0.58%**	100.00%	2.50	3.42	2.04	3.03
	slow-deep	–	–	**-**	0.429	1.53%	**0.66%**	100.00%	2.58	3.58	2.07	3.09

Abbreviations: n_i_, number of iterations; yrs, year.

**Fig. 4:**
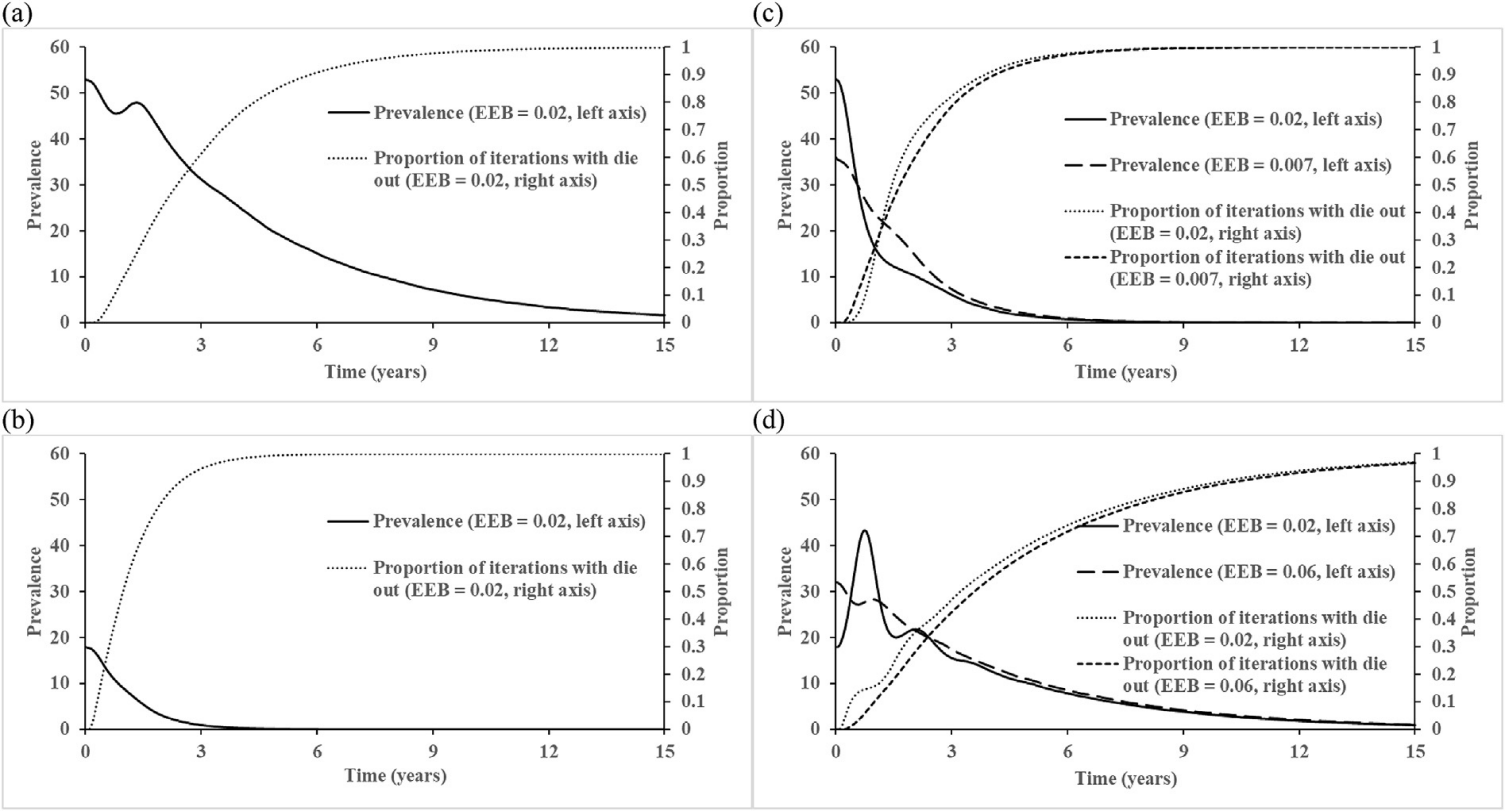
The effect of modifying the birth rate on the average total number of infected individuals in the populations with R_0_ = 20 and fast-shallow waning immunity dynamics: (a) N = 10,000, birth rate = 0.02; (b) N = 3,500, birth rate = 0.02; (c) N = 10,000, birth rates = 0.007 and 0.02 (used by Vallejo et al.); (d) N = 3,500, birth rates = 0.06 and 0.02 (used by Vallejo et al.) averaged over n_i_ = 100,000 over time. Abbreviations: EEB, endemic equilibrium birth rate.

Fig. 2, Fig. 3 (corresponding to Figures 4 and 5 of Vallejo et al. (2017)) show the total number of infectious people (first infection and reinfections) averaged over 1,000 iterations at every 100 event steps. Unlike the findings reported by Vallejo et al. (2017), we do not observe drastic fluctuations in the average number of infectious people, which represents a different behavior of our implementation of their model than they reported. Recognizing that the time between events differs in each iteration, which complicates interpretation of the x-axis in Fig. 2a–c, Fig. 2d–f shows the same results plotted on the more intuitive scale of time. Fig. 2d–f further show that circulation persists for the entire 15 years only in a very small fraction of the iterations, which we emphasize do not include any vaccination. Fig. 3, Fig. 4 show the impact of varying the birth rate on the 100 event step and natural time scales, respectively. Given that the birth rate may influence the length and strength of virus circulation, we suggest that the initial conditions for Fig. 3c–d and 4c-d should appropriately change with the introduction of new birth rates because the equilibrium changes (we note that Vallejo et al. (2017) did not change them), consequently leading to different starting points and shapes of the curves (see long-dashed lines). Thus, we suggest that analyses assumed to start at the endemic equilibrium should use the correct endemic equilibrium for the inputs so as to avoid the effects of the initial conditions representing a different point than the endemic equilibrium.

## 4 Discussion

Despite our efforts to carefully follow the model and algorithm in Vallejo et al. (2017), we could not reproduce their results of a relatively high probability of unusually long silent circulation of polioviruses in small populations. Although the two authors who independently reproduced the model obtained similar results to each other (within the stochastic variation), our results differ from the results reported by Vallejo et al. (2017) in a way that suggests some difference between the approaches that we could not identify based on the information provided in the published article or in follow up with the authors. Our use of more simulation runs and more iterations does not explain this difference. We found some similar general trends in the length of times between polio cases as a function of R_0_ or population size, but our analysis suggests much shorter times between polio cases and much lower probabilities of prolonged undetected circulation with perfect or sub-optimal case-based surveillance.

We also identified several issues with the presentation of the results by Vallejo et al. (2017). First, focusing in the abstract on the percentage of runs with at least three years between successive polio cases among iterations in which two or more polio cases occurred ignores the inflation implied by conditioning on the probability that circulation continues for long enough in a small population to generate at least two polio cases. While Tables 3 and 4 in Vallejo et al. (2017) provide the information required to get to the percentage of all runs with at least three years between successive polio cases, the reader must multiply the two numbers to obtain this result. Second, even with that adjustment, as noted above, the TBC metric does not represent the probability of circulation as a function of time without detections, which requires conditioning on the absence of detections. Eichner and Dietz (1996) developed a metric that truly informs the probability of circulation as a function of time without detections, which various other groups subsequently adopted to further address this question (Houy, 2015; Kalkowska, Duintjer Tebbens, Pallansch, et al., 2015). Third, the use of only 1,000 iterations remains insufficient to generate statistically robust findings for the relatively rare events that reflect the tails of highly skewed distributions. Fourth, the average prevalence of infections by 100 event steps remains challenging to interpret, because the time between events varies between individual stochastic iterations and depends strongly on the realized prevalence of infections. Results shown as a function of time (Fig. 2, Fig. 4) provide a more direct means for poliovirus epidemiologists to verify the plausibility of the findings, as does showing the behavior of individual realizations (Fig. 1).

Besides the questions about the numerical results and their presentation, the findings from Vallejo et al. (2017) remain limited by the simplifying assumptions of their hypothetical model. Despite the stated focus on investigating the silent circulation of poliovirus in small populations unreached by vaccination, the model ignores the heterogeneity and conditions that affect the ability of polioviruses to circulate in sub-populations and among different age groups. While low-level heterogeneity remains challenging to characterize despite its importance for die-out behavior, consideration of a more realistic aging process and different levels of isolation of an unvaccinated subpopulation within the generally well-vaccinated broader population may offer different insights. Furthermore, given that the results clearly depend on the assumed R_0_, we note the importance of the Vallejo et al. (2018) correction that emphasized that the R_0_ upper-bound of 20 does not produce results consistent with epidemiological observations in any realistic population. In our experience, the maximum R_0_ in the context of approximate fast-shallow waning equals 13 among a broad set of populations considered, including northern India (Duintjer Tebbens, Pallansch, Kalkowska, et al., 2013; Kalkowska, Duintjer Tebbens, Grotto, et al., 2015; Kalkowska, Duintjer Tebbens, & Thompson, 2014, 2014; Thompson, Kalkowska, & Duintjer Tebbens, 2015), although we note the dependence of R_0_ values on the model formulation. For an isolated, rural population in northern Nigeria, we suspect much lower R_0_ values (e.g., maximum of 10), which explains why we focused on presenting our results for the R_0_ value of 10. Thus, while we appreciate the importance of research that explores the robustness of assumptions, policy recommendations should draw from realistic models that represent the last WPV reservoirs. Moreover, poliovirus transmissibility varies seasonally, which implies greater fluctuations in the prevalence and leads to an increased probability of die-out during each low season compared to the Vallejo et al. (2017) model, which does not include seasonality. The assumption of small populations that remain untouched by vaccination for up to 15 years also remains highly questionable in the context of ongoing global polio eradication efforts and ignores the possible secondary OPV benefits derived from massive OPV use in the surrounding population. With almost all (>99.7%, see POE in Table 4) simulations resulting in eradication, the assumption of an endemic equilibrium state for perfectly isolated small populations does not appear realistic. This suggests the need to allow at least a small chance of transmission between sub-populations. In a situation of many small sub-populations but with some interaction among them, the chance that any individual sub-population sustains prolonged transmission without experiencing cases increases, which would imply a higher overall probability of silent circulation. However, such prolonged circulation would also imply an increased probability that the virus spreads to other subpopulations where it can cause cases and get detected, which decreases the overall probability of silent circulation. Examining the net effect of these counteracting trends remains a topic of further research. Lastly, the assumed single-stage process implies unrealistic proportions of infected individuals. The precise shape of the duration distribution remains uncertain and does not matter much for questions about vaccination threshold, equilibrium behavior, and cumulative incidence, but it substantially affects poliovirus persistence in small populations (Table 4).

Instead of concentrating solely on variation of the inputs using a model that simplifies much of the complexity related to poliovirus transmission, we suggest that future research of silent circulation in small populations should focus on realistic model structures and model inputs representative of the last WPV reservoirs.

## Declarations of interest

None.
